# Analysis of BIS and Patient State Index in Children Undergoing General Anesthesia

**DOI:** 10.1111/pan.70063

**Published:** 2025-09-30

**Authors:** Zaccaria Ricci, Denise Colosimo

**Affiliations:** ^1^ Department of Emergency and Critical Care, Anesthesia and Pediatric Intensive Care Unit Meyer Children's Hospital, IRCCS Florence Italy; ^2^ Department of Health Science, Section of Anesthesia and Intensive Care University of Florence Florence Italy

**Keywords:** depth of anesthesia, neurodevelopment, neuromonitoring, processed electroencephalography

1

Electro‐encephalographic (EEG) guided anesthesia both in adult [1] and pediatric patients [2] has shown a reduction of anesthetic dose and less postoperative delirium [3]. Typically, processed EEG (pEEG) is utilized, a quantitative analysis in which dimensionless‐derived anesthesia EEG indices are computed into a single value. These algorithms do not account for age‐related physiological changes in the EEG, considering that frontal alpha activity begins to emerge after the 4th month of life and frontal alpha power peaks at 7 years old (yo) [4]. Significant differences have been reported between pEEG values of patients younger than 2 years old compared to older patients undergoing similar levels of hypnosis depth [5, 6].

The aim of this study was to compare the pEEG values (i.e., BIS and Patient State Index ‐ PSI) simultaneously derived by two different systems in pediatric patients older than 2 years, undergoing inhalational anesthesia.

We conducted a secondary analysis of a prospective observational study [7]. The study was approved by the Hospital Ethics Committee (Comitato Etico Regionale Pediatrico per la Sperimentazione Clinica della Regione Toscana, n. 310/2022). Methods are described in the File S1.

From the 51 patients enrolled in the previous study, we excluded 4 patients undergoing total intravenous anesthesia and 4 patients below 2 years old. Table 1 presents the baseline and demographic data of the 43 analyzed children: of these, 28 were in the age group 2–7 and 25 in the group 8–15.

**TABLE 1:** General demographics and baseline data.

Pts n.	43
Age (years)	8.5 (4–10.5)
Weight (Kg)	29 (18–43)
Surgery duration (min)	105 (70–162)
Heart rate (bpm)	88 (76–115)
Mean arterial pressure (mmHg)	69 (58–85)
ASA (score: pts. n.)	1: 22 2: 18 3: 3
Surgery (type: pts. n.)	General surgery = 7 Head (ENT and oculist) = 4 Orthopedics: 22 Urology: 6 Other: 4

*Note:* Absolute numbers (n.) or median (interquartile range).

Abbreviations: ASA, American Society of Anesthesiology; ENT, ear, nose, and throat; LMA, laryngeal mask airway; Pts: patients.

A total of 387 BIS/PSI couples were analyzed. Nonlinear correlation was shown in the overall sample (Figure 1), with an *r* ^2^ of 0.86. No relevant differences were found in the subgroup 2–7 years old (*r* ^2^ 0.87) vs. the subgroup 8–15 years old (*r* ^2^ 0.86). The median values over the analyzed time points were coherent with the reference values recommended by the two devices in the surgical procedure phases (Figure S1). The percentage modifications of the BIS and PSI values from one time point to the following were calculated, and we found no significant differences (BIS‐PSI) in the percentage increase or reduction between the two monitors (*p* = 0.84). Overall, mean percentage modification differences were −1.83 and 95% CI from −10.4 to 6.7; they appeared smaller during the maintenance phase (1.7, 95% CI −5.4 to 9), whereas in the loss of consciousness and recovery of consciousness phases, variations appeared greater (mean difference −13.7, 95% CI −68 to 40).

**FIGURE 1:**
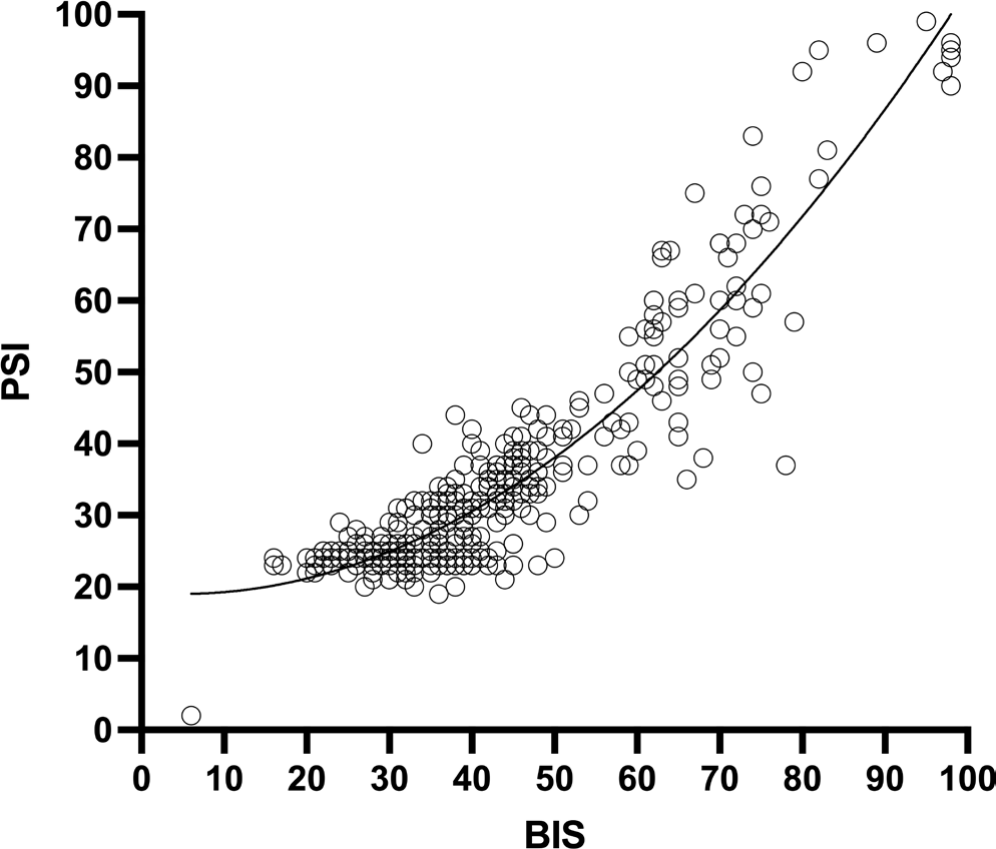
Correlation between BIS and PSI. The nonlinear curve describes that at lower BIS values (in the range of 20–45), the increase in PSI is small (20–30), whereas at higher BIS and PSI values the correlation curve is steeper.

The key findings of this study are as follows: (1) In children between 2 and 15 years, BIS and PSI values appeared strongly correlated in a nonlinear fashion. (2) In our patients, BIS and PSI values appeared coherent with the surgical/anesthetic phase undergoing inhalational anesthesia. (3) pEEG values showed greater percentage differences in the induction and recovery phases.

Previous studies comparing BIS and PSI in children receiving inhalational anesthesia showed controversial results. Kim and coworkers showed a weak correlation and concluded that these monitors are not reliable in pediatric patients [8]. In particular, in the age group from 2 to 7 years they showed that BIS and PSI had no correlation, with one value increasing when the other was not changing when sevolflurane dose was increased. Differently Jang and coworkers showed, in a cohort of children 3–12 years old, “relatively good agreement” between the two monitors and concluded that they are appropriate for monitoring for different state of anesthesia in the pediatric population [9].

Our study confirms that BIS and PSI algorithms in patients older than 2 years undergoing inhalational anesthesia may be considered comparable. From a neurophysiological standpoint, it can be speculated that EEG activity of patients above 2 years can be considered “mature” enough for pEEG algorithms. Since the recommended ranges of the two devices indicate a deep anesthetic level at 25–50 in the SedLine monitor and 40–60 in the BIS, this might be the reason why PSI% modifications from the awake state (around 80–100 in both monitors) appear greater than BIS% modifications (more negative difference). Interestingly, we observed two different patterns in the nonlinear correlation curve: at lower BIS values (in the range of 20–45), the increase in PSI is small (20–30), whereas at higher BIS and PSI values the correlation curve is steeper. The clinical value of this observation is uncertain, but it describes how the algorithms work at deeper anesthetic states.

This study has some limitations. First, the sample size was selected arbitrarily and derived as a post hoc analysis from a previous observational study. Second, we conducted manual data collection to simulate real‐world EEG monitoring by anesthesiologists. Automated data download minimizes observer error and enhances temporal resolution, ensuring that rapid changes are accurately captured. Third, sensor positioning might have affected the accuracy of the monitoring systems. Fourth, we did not evaluate the role of artifacts, and if they affected the two monitors differently, since we did not analyze raw traces. Finally, the direct correlation of proprietary processed values may overstate clinical equivalence and should not be interpreted, based on our findings, as the monitors are definitely interchangeable.

BIS and PSI are non‐linearly correlated in children between 2 and 15 years undergoing inhalational anesthesia. These results should be further confirmed, and clinicians must implement these correlation measures with constant clinical judgment to avoid overinterpretation of statistical associations.

## Conflicts of Interest

The authors declare no conflicts of interest.

## Supporting information


**Figure S1:** Median (interquartile range) of BIS and patient state index (PSI) values over the predefined time points.

**Data S1:** pan700663‐sup‐0002‐Supinfo.docx.

## Data Availability

The data that support the findings of this study are available from the corresponding author upon reasonable request.
