# Supplementary material for: Analysis of BIS and Patient State Index in Children Undergoing General Anesthesia
Source: Paediatr Anaesth. 2025 Sep 30;36(1):104–6. doi: 10.1111/pan.70063 (PMC12686747; doi:10.1111/pan.70063)
Supplement: Supplementary file 1 — Figure S1: Median (interquartile range) of BIS and patient state index (PSI) values over the predefined time points. [file PAN-36-104-s001.docx]

**Supplementary figure 1.** Median (interquartile range) of BIS and patient state index (PSI) values over the predefined time points.
