# Supplementary material for: Analysis of BIS and Patient State Index in Children Undergoing General Anesthesia
Source: Paediatr Anaesth. 2025 Sep 30;36(1):104–6. doi: 10.1111/pan.70063 (PMC12686747; doi:10.1111/pan.70063)
Supplement: Supplementary file 2 — Data S1: pan700663‐sup‐0002‐Supinfo.docx. [file PAN-36-104-s002.docx]

**Methods**

SedLine® (Masimo, Irvine, CA, USA) and BIS® (Medtronic, Dublin, Ireland) monitors were used simultaneously in each patient. SedLine’s and BIS’s sensors were both placed in patients’ forehead in order to obtain the recording of the two values at the same time points. BIS and PSI values were recorded at induction, during laryngeal mask or endotracheal tube placement, at surgical incision, and every 15 minutes (min) thereafter until the end of surgery, including extubation and discharge from the operating room. Written informed consent was obtained from parents or legal guardians. Children aged 2 to 15 years and cases undergoing inhalational anesthesia were retrieved from the original study. Strengthening the Reporting of Observational Studies in Epidemiology (STROBE) guidelines were applied.

Demographic data, including diagnosis, American Society of Anesthesia (ASA) score, surgical procedure were also recorded. Anesthesia was administered as inhalational anesthesia targeting MAC_age_ of 1, unless clinical judgement required to increase or lower anesthetic dose. Fentanyl at boluses of 1 mcg/kg were administered at induction and upon anesthesiologist decision. Anesthesia was not pEEG guided.

The percentage modifications of the BIS and PSI values from one time point to the following were calculated as follows: [BIS or PSI value at time point x+1/ BIS or PSI value at time point x]-100. I.e. if BIS changes from 50 to 40 the percentage change is [40/50*100]-100= -20%. This represents the percentage reduction (if negative) or increase (if positive) of the pEEG values at successive time points. We hypothesized they should be similar with the two systems. Eventually, the differences between the percentage modifications of BIS and PSI were also calculated. The smallest the difference, the closest the percentage modification between the two monitors.

The primary aim of our prospective observational study was to determine the correlation between BIS and PSI values. Secondary aims included assessing absolute values over time and if the percentage modification of pEEG values over time matched between the monitors. Furthermore, we explored if any difference was present in patients below and above 7 years old.

All data are presented as median (interquartile range) or mean (95% confidence interval -CI-) were used as appropriate. The Wilcoxon test for paired measures was used to assess differences between PSI and BIS. Nonlinear regression (r²) was used to verify the association between continuous variables. A p-value of <0.05 was considered statistically significant. Statistical analysis was performed using the GraphPad Prism 10.0 software package (GraphPad Software, San Diego, CA, USA).
